# The effect of critical coupling constants on superconductivity enhancement

**DOI:** 10.1038/s41598-023-33809-5

**Published:** 2023-04-20

**Authors:** Peir-Ru Wang, Jien-Wei Yeh, Yi-Hsien Lee

**Affiliations:** Department of Materials Science and Engineering, National Tsing Hua University, 30013 Hsinchu, Taiwan

**Keywords:** Superconducting properties and materials, Superconducting properties and materials

## Abstract

In this study, we propose a phenomenological model to extend McMillan's results on a coupling strength equal to 2. We investigate possible strategies to enhance superconductivity by tuning the phonon frequency, carrier number, or pressure. In particular, we show that the critical coupling constants corresponding to the phonon frequency, carrier number, or pressure determine whether the variation of the critical temperature is positive or negative. These observations explain the contrasting behavior between weak and strong coupling superconductors and are consistent with experimental observations. We also demonstrate the dome observed in the carrier number effect and pressure effect. Additionally, these critical coupling constants systematically separate superconductivity into three regions: weak, intermediate, and strong coupling. We find that the enhancement strategies for weak and strong coupling regions are opposite, but both inevitably bring superconductivity into the intermediate coupling region. Finally, we propose general zigzag methods for intermediate coupling superconductors to further enhance the critical temperature.

## Introduction

Notably, increasing the superconducting critical temperature ${T}_{c}$ remains the principal problem of condensed matter physics since the discovery of superconductivity^1^. Specifically, varying the phonon spectrum, tuning the carrier number, and increasing the pressure are three important experimental approaches to enhance ${T}_{c}$ and consequently, speculate possible theories of superconductivity. However, the effects of altering these parameters on ${T}_{c}$ are quite contrasting between weak coupling (low ${T}_{c}$) and strong coupling (high ${T}_{c}$) superconductivity. More precisely, first, in metallic superconductors^2–6^ and nickel-based superconductors^7^, which correspond to the case of weak coupling, the critical temperature ${T}_{c}$ can be increased via phonon softening. In McMillan's results^3^, the maximum ${T}_{c}$ appeared when the coupling strength is equal to 2. In contrast, in cuprate systems, which correspond to the case of strong coupling, ${T}_{c}$ can be increased via phonon stiffening^8,9^. Second, the effect of pressure on ${T}_{c}$ is negative in most metallic superconductors^10,11^. On the other hand, positive effects or a dome-like delineation appear in cuprate systems^11,12^, iron-based^13,14^, and hydrogen-rich superconductors^15,16^. Third, in the phase diagrams that illustrate the variation of ${T}_{c}$ with respect to the carrier number, the doom-like delineation may be observed in metallic^17,18^, cuprates^19–21^, and iron-based superconductors^22^. Specifically, the underdoped region is strongly coupled with a positive carrier number effect and becomes weakly coupled in the overdoped region with a negative carrier number effect^23^. Besides, another approach to increase the carrier number is to gate thin film materials, which also demonstrates the dome-like effect^24–26^. These three phenomena have a common dome-like delineation—the positive tendency appears in strong coupling superconductivity, and becomes negative in weak coupling superconductivity.

Furthermore, the ${T}_{c}$ relation that derives from Cooper instability is a general property of superconductivity. Specifically, this relation may be written in the form ${T}_{c}\sim \mathcal{W}\mathrm{exp}\left(-{\lambda }^{-1}\right)$^2,27,28^, where $\mathcal{W}$ is the bandwidth of superconducting electrons, and $\lambda$ is the coupling constant of the pairing. Conventional superconductors, including metallic, MgB_2_^29^, and hydrogen-rich compounds, are adequately explained by phonon-mediated pairing^2–4,16^. Meanwhile, unconventional superconductors, such as cuprates and iron-based superconductors are explained by Hubbard-type theories^30–34^. Moreover, although the strong repulsion between electrons plays a major role in the Hubbard model, the electron–phonon interaction remains nonnegligible and consequently, contributes to the unconventional superconductivity^21,35,36^. For this reason, in this study, we discuss the effects of varying the phonon spectrum, the carrier number, and the pressure on phonon-mediated pairing.

In the phonon-mediated pairing, the bandwidth $\mathcal{W}$ is the characteristic phonon frequency $\Omega$, and the coupling constant $\lambda =g\left({E}_{F}\right){V}_{eff}$ is the product of the effective interaction between electrons ${V}_{eff}$ and the density of state at Fermi level $g\left({E}_{F}\right)$. The effective interaction ${V}_{eff}$ based on the Migdal theory^37^ is ${V}_{eff}\sim 1/\left(M{\Omega }^{2}\right)$, and the density of state at Fermi level^38^ $g\left({\varepsilon }_{F}\right)={m}^{*}\sqrt[3]{3{\pi }^{2}Z{n}_{ion}}/{\pi }^{2}{\mathrm{\hslash }}^{2}$, where $M$ is the ion mass, ${m}^{*}$ is the effective mass of an electron, $Z$ is the valence number, and ${n}_{ion}$ is the ion number density. Therefore, the explicit form of $\lambda$ and *T*_*c*_ are

1 $$\lambda \left(\Omega \right)=\frac{C \sqrt[3]{Z{n}_{ion}}}{M{\Omega }^{2}}$$

and

2 $${T}_{c}\left(\Omega \right)\sim \mathrm{\Omega exp}\left[-\frac{M{\Omega }^{2}}{C\sqrt[3]{Z{n}_{ion}}}\right],$$

where $C$ is a constant. Notice that Eq. (1) is similar to McMillan^3^. However, we preserve the term $\sqrt[3]{Z{n}_{ion}}$ to describe the carrier number effect and the pressure effect. First, to investigate the phonon effect on superconductivity, the derivative of Eqs. (1) and (2) with respect to $\Omega$ are

3 $$\frac{d\lambda }{d\Omega }=-2\frac{\lambda }{\Omega }$$

and

4 $$\frac{d{T}_{c}}{d\Omega }=\frac{{T}_{c}}{\Omega }\left(1-\frac{2}{\lambda }\right).$$

The derivative $d{T}_{c}/d\Omega$ is equal to 0 when $\lambda$ is equal to 2. Define the critical coupling constant corresponding to the phonon frequency ${\lambda }_{c}^{\Omega }$=2.

Second, to investigate the dependency of $\lambda$ and ${T}_{c}$ on carrier number $Z$, Eqs. (1) and (2) can be rewritten as a function of the carrier number $Z$. Here, the characteristic phonon frequency $\Omega$ uses the jellium phonon frequency^39^ $\Omega =\sqrt{{\mathrm{Z}}^{2}{e}^{2}{n}_{ion}/{\epsilon }_{0}M}$, where ${\epsilon }_{0}$ is the permittivity. The explicit form of $\lambda$ and ${T}_{c}$ as a function of $Z$ are

5 $$\lambda \left(Z\right)=\frac{C{\epsilon }_{0}}{{e}^{2}{n}_{ion}^{2/3}}{Z}^{-\frac{5}{3}}$$

and

6 $${T}_{c}\left(Z\right)\sim {\left(\frac{{\mathrm{Z}}^{2}{e}^{2}{n}_{ion}}{{\epsilon }_{0}M}\right)}^\frac{1}{2}\mathrm{exp}\left(-\frac{C{\epsilon }_{0}}{{e}^{2}{n}_{ion}^{2/3}}{Z}^{-\frac{5}{3}}\right).$$

The derivative of Eqs. (5) and (6) with respect to $Z$ are

7 $$\frac{d\lambda }{dZ}=-\frac{5}{3}\frac{\lambda }{Z}$$

and

8 $$\frac{d{T}_{c}}{dZ}=\frac{{T}_{c}}{Z}\left[1-\frac{\left(\frac{5}{3}\right)}{\lambda }\right].$$

Similarly, the derivative $d{T}_{c}/dZ$ is equal to 0 when $\lambda$ is equal to 5/3, denoted as ${\lambda }_{c}^{Z}$.

Third, to investigate the dependency of ${T}_{c}$ on pressure $P$, the compressibility $\beta =-\left(1/V\right)*\left(dV/dP\right)$ can be adopted to relate the pressure $P$ and the volume $V$. Using ${n}_{ion}={N}_{ion}/V$, where ${N}_{ion}$ is the number of ions; Eqs. (5) and (6) can thus be rewritten into a function of $V$:

9 $$\lambda \left(V\right)=\frac{C{\epsilon }_{0}}{{e}^{2}{Z}^\frac{5}{3}{N}_{ion}^\frac{2}{3}}{V}^\frac{2}{3}$$

and

10 $${T}_{c}\left(V\right)\sim {\left(\frac{{Z}^{2}{e}^{2}{N}_{ion}}{{\epsilon }_{0}MV}\right)}^\frac{1}{2}\mathrm{exp}\left(-\frac{{e}^{2}{Z}^\frac{5}{3}{N}_{ion}^\frac{2}{3}}{C{\epsilon }_{0}}{V}^{-\frac{2}{3}}\right).$$

The derivative of Eqs. (9) and (10) with respect to the pressure $P$ are:

11 $$\frac{d\lambda }{dP}=\frac{dV}{dP}*\frac{d\lambda }{dV}=-\beta V*\frac{2}{3}\frac{\lambda }{V}$$

and

12 $$\frac{d{T}_{c}}{dP}=\frac{dV}{dP}*\frac{d{T}_{c}}{dV}=\beta V*\frac{1}{2}\frac{{T}_{c}}{V}\left[1-\frac{\left(\frac{4}{3}\right)}{\lambda }\right].$$

**Figure 1:**
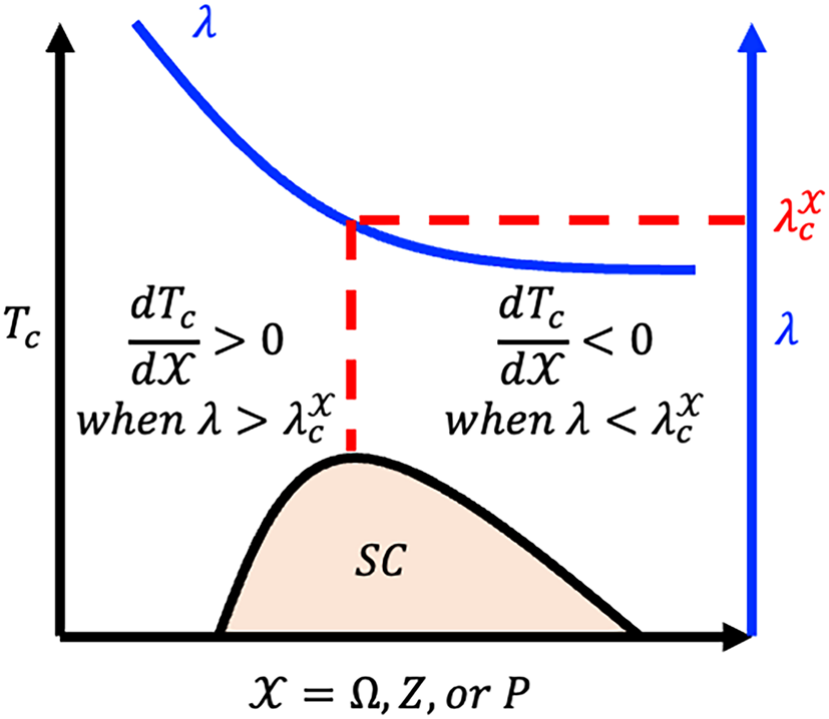
A schematic diagram of the critical temperature ${T}_{c}$ and the critical coupling constants ${\lambda }_{c}^{\mathcal{X}}$ corresponding to the phonon frequency $\Omega$, the carrier number $Z$, and the pressure $P$. There is a sign change of derivative $d\lambda /d\mathcal{X}$ at ${\lambda }_{c}^{\mathcal{X}}$. If $\lambda$ is larger than ${\lambda }_{c}^{\mathcal{X}}$, the effect of varying $\mathcal{X}$ on superconductivity is positive with ${T}_{c}$ increasing corresponding to strong coupling superconductivity. Meanwhile, if $\lambda$ is smaller than ${\lambda }_{c}^{\mathcal{X}}$, then the effect of varying $\mathcal{X}$ on superconductivity is negative with ${T}_{c}$ decreasing corresponding to the weak coupling superconductivity.

The critical coupling constant corresponding to the pressure is ${\lambda }_{c}^{P}$=4/3. The schematic diagram based on the result from Eqs. (1) to (12), which demonstrates the effects of tuning $\Omega$, $Z$, or $P$ on ${T}_{c}$ is plotted in Fig. 1.

## Results and discussion

### The origin of the dome

The results of our derivations reveal two common features: (1) $d{T}_{c}/d\mathcal{X}\propto 1-\left({\lambda }_{c}^{\mathcal{X}}/\lambda \right)$, and (2) $d\lambda /d\mathcal{X}<0$, where $\mathcal{X}$ represents $\Omega$, $Z$, or $P$. Specifically, feature (1) indicates that there is a sign change of derivative $d\lambda /d\mathcal{X}$ at ${\lambda }_{c}^{\mathcal{X}}$. If $\lambda$ is larger than ${\lambda }_{c}^{\mathcal{X}}$, the effect of varying $\mathcal{X}$ on superconductivity is positive with ${T}_{c}$ increasing, which corresponds to strong coupling superconductivity. On the other hand, if $\lambda$ is smaller than ${\lambda }_{c}^{\mathcal{X}}$, then the effect of varying $\mathcal{X}$ on superconductivity is negative with ${T}_{c}$ decreasing, which corresponds to weak coupling superconductivity. These observations explain the contrasting behavior between weak and strong coupling superconductors. Feature (2) is the reason for the dome-like delineation observed in many strong coupling superconductors. Specifically, for strong coupling superconductors, the coupling constant is expected to be larger than ${\lambda }_{c}^{\mathcal{X}}$, to ensure the effect of varying $\mathcal{X}$ on superconductivity is positive. In addition, since the derivative of $\lambda$ is negative, $\lambda$ decreases when the parameter $\mathcal{X}$ increases. In particular, once $\lambda$ becomes smaller than ${\lambda }_{c}^{\mathcal{X}}$, the effect of varying $\mathcal{X}$ on superconductivity becomes negative. This transition from positive to negative demonstrates the dome-like delineation and can be observed when tuning the carrier number or varying the pressure in the experiments.

### Critical temperature ${{\varvec{T}}}_{{\varvec{c}}}$ as a function of phonon frequency ${\varvec{\Omega}}$

Critical temperature ${T}_{c}$ is influenced when the phonon frequency $\Omega$ is changing. Specifically, the maximum ${T}_{c}$ appears at ${\lambda }_{c}^{\Omega }$ = 2 by varying the phonon frequency Ω, which is consistent with McMillan^3^. More precisely, in the $\lambda >{\lambda }_{c}^{\Omega }$ region, the sign of $d{T}_{c}/d\Omega$ is positive, and ${T}_{c}$ increases when increasing $\Omega$. This region may correspond to cuprate superconductors. Particularly, LaBaCuO, YBaCuO, BiSrCaCuO, HgBaCaCuO, and TlBaCaCuO demonstrate phonon stiffening effect^8,9^. In Table 1, the $\lambda$ value of phonon stiffening effect superconductors are listed. For example, the value of the coupling constant $\lambda$ at optimal ${T}_{c}$ of Bi_2_Sr_2_Ca_0_Cu_1_O_x_, Bi_2_Sr_2_Ca_1_Cu_2_O_x_, and Bi_2_Sr_2_Ca_2_Cu_3_O_x_ are 2.95, 2.15, and 2.18, respectively^21^. These $\lambda$ values are larger than ${\lambda }_{c}^{\Omega }$ indicating that ${T}_{c}$ can be enhanced by the phonon stiffening effect.

**Table 1:** Examples of phonon effect $\Omega$ on superconductivity and $\lambda$ value.

Compound	$\lambda$
Phonon stiffening	
Bi_2_Sr_2_Ca_0_Cu_1_O_x_	2.95^21^
Bi_2_Sr_2_Ca_1_Cu_2_O_x_	2.15^21^
Bi_2_Sr_2_Ca_2_Cu_3_O_x_	2.18^21^
YBa_2_Cu_3_O_x_	2.32^40^
Phonon softening	
VCr	0.53^3^
ZrRh	0.80^3^
NbMo	0.7^3^
MoRe	0.86^3^
WRe	0.60^3^
PbTl	1.53^4^
PbBi	1.66^4^
Nb_3_Al	1.2–1.82^5,41^
Nb_3_Ge	0.7–1.5^6^
BaNi_2_As_2_	0.16–0.24^42^

The critical coupling constant corresponding to the phonon frequency is ${\lambda }_{c}^{\Omega }$ = 2. The phonon stiffening effect appears when $\lambda >{\lambda }_{c}^{\Omega }$, and the phonon softening effect appears when $\lambda <{\lambda }_{c}^{\Omega }$.

Meanwhile, in the $\lambda <{\lambda }_{c}^{\Omega }$ region, the coupling constant $\lambda$ is smaller than ${\lambda }_{c}^{\Omega }$. Specifically, the sign of $d{T}_{c}/d\Omega$ is negative and ${T}_{c}$ decreases when $\Omega$ increases. More precisely, this region may correspond to traditional metallic superconductors. Particularly, VCr, ZrRh, NbMo, MoRe, WRe, PbTl, PbBi, Nb_3_Al, and Nb_3_Ge demonstrate the phonon softening effect^3–6^. In Table 1, the $\lambda$ value of phonon softening effect superconductors are listed. For example, when the Debye temperature ${\Theta }_{\mathrm{D}}$ of the VCr alloy increases from 370 to 470 K, ${T}_{c}$ decreases from 3.21 to 0.10 K, and $\lambda$ decreases from 0.53 to 0.33. Taking another example, when the Debye temperature ${\Theta }_{\mathrm{D}}$ of the ZrRh alloy increases from 192 to 244 K, ${T}_{c}$ decreases from 5.95 to 3.10 K, and $\lambda$ decreases from 0.80 to 0.59. The experiment data show that the coupling constant $\lambda$ decreases when the phonon frequency increases, which is consistent with our result $d\lambda /d\Omega <0$ from Eq. (3). A similar effect also appears in nickel-based superconductors^7^, which validates that the superconductivity is enhanced by giant phonon softening.

### Critical temperature ${{\varvec{T}}}_{{\varvec{c}}}$ as a function of carrier number ${\varvec{Z}}$

The effect of carrier number on superconductivity through varying the alloy composition, doping concentration, or gating voltage has been widely studied in material physics. Particularly, the dome-like delineation was observed in metallic materials^17^, cuprates^19–21^, iron-based systems^22^, and gating thin film materials^24^. More precisely, the maximum ${T}_{c}$ appears at ${\lambda }_{c}^{\mathrm{Z}}$ = 5/3 by varying the carrier number $Z$.

**Figure 2:**
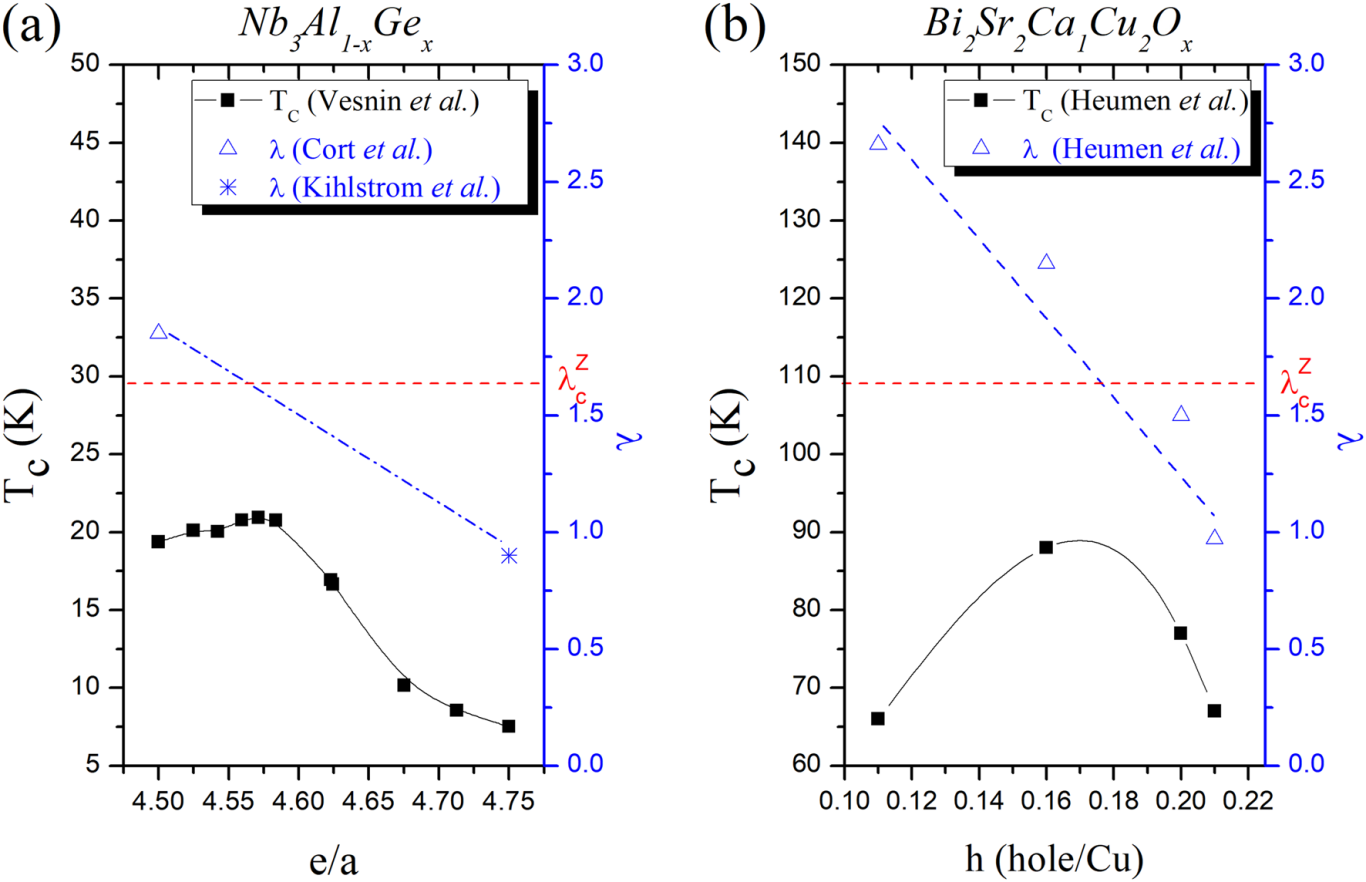
Examples of carrier number effect $Z$ on superconductivity. (**a**) The conventional superconductors Nb_3_Al_1−x_Ge_x_ demonstrates carrier effect^5,6,41,43^. Specifically, the electrons per atom ratio (e/a) rises from 4.50 to 4.75 and ${T}_{c}$ shows dome-like effect. (**b**) The hole-doped h (holes/Cu) experiment of the unconventional superconductors Bi_2_Sr_2_Ca_1_Cu_2_O_x_ demonstrates dome-like effect^21^. The critical coupling constant corresponding to $Z$ is ${\lambda }_{c}^{Z}$ = 5/3. Both cases show that ${T}_{c}$ is increasing when $\lambda >{\lambda }_{c}^{Z}$, and ${T}_{c}$ is decreasing when $\lambda <{\lambda }_{c}^{Z}$.

For conventional superconductors, we take Nb_3_Al_1−x_Ge_x_ as an example^43^, which is showing in Fig. 2a. Specifically, when the Ge component rises from x = 0 to x = 0.29, the electrons per atom ratio (e/a) rises from 4.50 to 4.57 and ${T}_{c}$ increases from 18 to 21 K. The coupling constant of Nb_3_Al_1_ is $\lambda$ > 1.8^41^, which is greater than ${\lambda }_{c}^{Z}$ and is consistent with the positive carrier effect. Moreover, when the Ge component rises from x = 0.29 to x = 1, the e/a ratio rises from 4.57 to 4.75 and ${T}_{c}$ decreases from 21 to 7 K. The coupling constant of Nb_3_Ge_1_ is $\lambda$ < 1^6^ which is smaller than ${\lambda }_{c}^{Z}$ and is consistent with the negative carrier effect.

For unconventional superconductors, we take the hole-doped experiment of Bi_2_Sr_2_Ca_1_Cu_2_O_x_ as an example^21^, which is showing in Fig. 2b. Specifically, when the hole doping h (holes/Cu) rises from h = 0.11 to h = 0.21, the coupling constant monotonically decreases from $\lambda$ = 2.66 to $\lambda$ = 0.97. Additionally, the critical temperature first rises from 66 K ($\lambda$ = 2.66 > ${\lambda }_{c}^{Z}$ at h = 0.11) to 88 K ($\lambda$ = 2.15 > ${\lambda }_{c}^{Z}$ at h = 0.16), then drops to 77 K ($\lambda$ = 1.5 < ${\lambda }_{c}^{Z}$ at h = 0.20) and further to 67 K ($\lambda$ = 0.97 < ${\lambda }_{c}^{Z}$ at h = 0.21). This dome-like delineation showed that the superconductivity is increasing in the underdoped region with coupling $\lambda$ stronger than ${\lambda }_{c}^{Z}$, and decreasing in the overdoped region with coupling $\lambda$ weaker than ${\lambda }_{c}^{Z}$.

### Critical temperature ${{\varvec{T}}}_{{\varvec{c}}}$ as a function of pressure effect ${\varvec{P}}$

The effect of pressure on superconductivity is an important topic in the field of condensed matter physics. In particular, for strong coupling superconductors, the pressure effect is positive and may lead to a dome-like delineation at higher pressure. These characteristics were observed in cuprate superconductors^12,44^, iron-based superconductors^13,14,45^, and hydrogen-rich superconductors^15,16^. In Table 2, the $\lambda$ value of positive or dome-like pressure effect superconductors are listed. Specifically, the coupling constants of cuprates are greater than ${\lambda }_{c}^{P}$ as mentioned in the previous section. Here, we note the couple constant of iron-based superconductors; for instance, LaFeAsO_1−x_F_x_ and Ba(Fe_1−x_Co_x_)_2_As_2_ are $\lambda$ = 2.38 and 2.83, respectively^46^. These strong coupling superconductors have coupling strength $\lambda$ larger than ${\lambda }_{c}^{P}$, thus the positive or dome-like delineation of the pressure effect can be observed.

**Table 2:** Examples of pressure effect $P$ on superconductivity and $\lambda$ value.

Compound	$\lambda$
Positive or dome-like	
Bi_2_Sr_2_Ca_0_Cu_1_O_x_	2.95^21^
YBa_2_Cu_3_O_x_	2.32^40^
LaFeAsO_1−x_F_x_	2.38^46^
Ba(Fe_1−x_Co_x_)_2_As_2_	2.83^46^
FeSe	1.6^47^
H_3_S	~ 2^48^
Negative	
Al	0.38^3^
Cd	0.38^3^
Sn	0.6^3^
In	0.69^3^
Pb	1.12^3^
MgB_2_	0.7^49^

The critical coupling constant corresponding to the pressure is ${\lambda }_{c}^{P}$ = 4/3. The positive or dome-like pressure effect appears when $\lambda >{\lambda }_{c}^{P}$, and the negative pressure effect appears when $\lambda <{\lambda }_{c}^{P}$.

Meanwhile, most metallic superconductors are weak-coupling superconductors. Particularly, Al, Cd, Sn, In, and Pb are examples with negative pressure effect^10,11^. Specifically, the coupling constant $\lambda$ of Al, Cd, Sn, In, and Pb are less than ${\lambda }_{c}^{P}$ (see Table 2). Clearly, these superconductors have $\lambda$ less than ${\lambda }_{c}^{P}$. Additionally, the negative pressure effect is also observed in the covalent compound MgB_2_^11^, with $\lambda$ equal to 0.7^49^, which is smaller than ${\lambda }_{c}^{P}$.

### The strategies of ${{\varvec{T}}}_{{\varvec{c}}}$ enhancement

**Figure 3:**
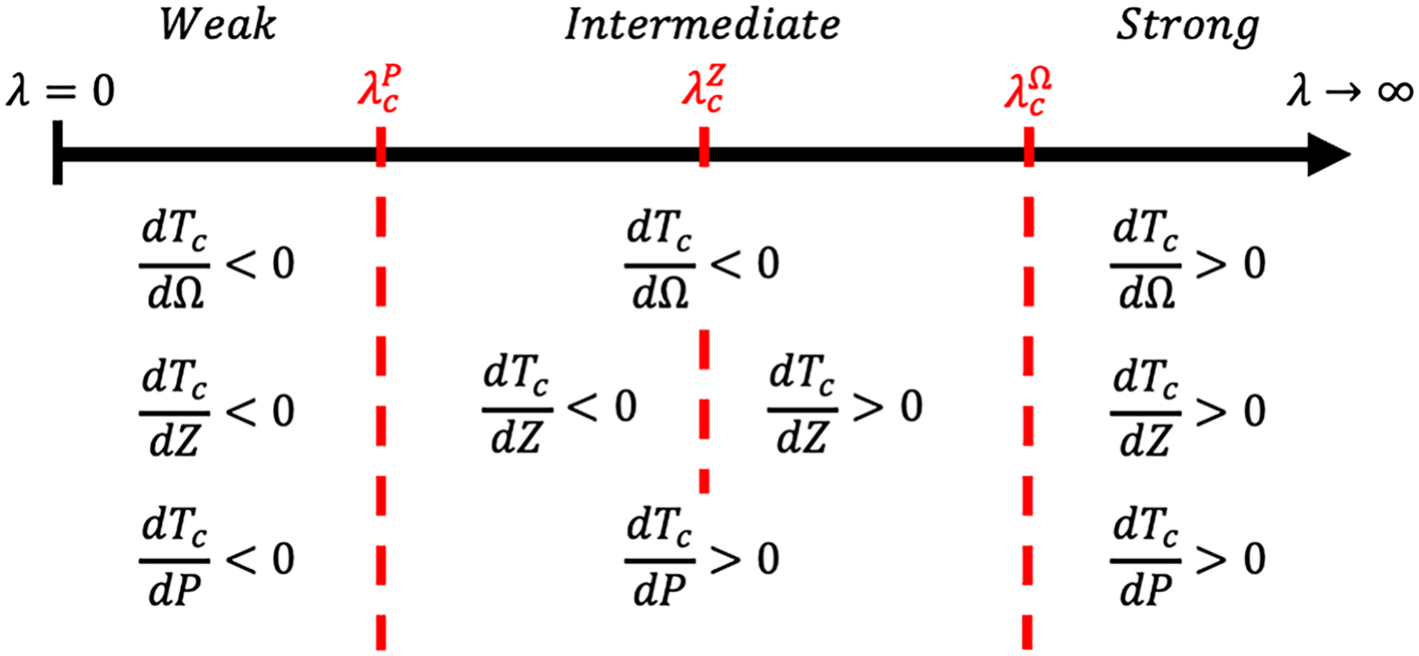
Classification and enhancement strategy for superconductivity. Using three critical coupling constants: ${\lambda }_{c}^{\Omega }$, ${\lambda }_{c}^{Z}$, and ${\lambda }_{c}^{P}$, superconductors can be classified by their coupling strength: weak coupling ($\lambda <{\lambda }_{c}^{P}$), intermediate coupling (${\lambda }_{c}^{P}<\lambda <{\lambda }_{c}^{\Omega }$), and strong coupling ($\lambda >{\lambda }_{c}^{\Omega }$).

According to these three critical coupling constants: ${\lambda }_{c}^{\Omega }$, ${\lambda }_{c}^{Z}$, and ${\lambda }_{c}^{P}$, superconductors can be classified by their coupling strength: weak coupling ($\lambda <{\lambda }_{c}^{P}$), intermediate coupling (${\lambda }_{c}^{P}<\lambda <{\lambda }_{c}^{\Omega }$), and strong coupling ($\lambda >{\lambda }_{c}^{\Omega }$). A schematic diagram can be seen in Fig. 3.

In the weak coupling region ($\lambda <{\lambda }_{c}^{P}$), the coupling constant $\lambda$ is less than ${\lambda }_{c}^{P}$. Particularly, all derivatives of critical temperature $d{T}_{c}/d\Omega$, $d{T}_{c}/dZ$, and $d{T}_{c}/dP$ are negative, as shown in Fig. 3. Hence, by decreasing $\Omega$, $Z$ or $P$, the superconductivity can be enhanced. These procedures increase the coupling constant $\lambda$, because the derivatives of the coupling constant $d\lambda /d\Omega$, $d\lambda /dZ$, and $d\lambda /dP$ are negative. We can accurately infer that the superconductivity of weak coupling superconductors is enhanced by increasing the coupling strength. In addition, once the coupling constant $\lambda$ becomes greater than ${\lambda }_{c}^{P}$, the superconductivity enters into the intermediate region, and the tendencies of ${T}_{c}$ become complicated.

Meanwhile, in the strong coupling region ($\lambda >{\lambda }_{c}^{\Omega }$), the coupling constant $\lambda$ is larger than ${\lambda }_{c}^{\Omega }.$ Particularly, all derivatives of the critical temperature $d{T}_{c}/d\Omega$, $d{T}_{c}/dZ$, and $d{T}_{c}/dP$ are positive in Fig. 3, thus the superconductivity can be increased by increasing $\Omega$, $Z$ or $P$. These procedures decrease the coupling constant $\lambda$, such that we can adequately infer that the superconductivity of strong coupling superconductors is enhanced by decreasing the coupling strength. Once the coupling constant $\lambda$ is smaller than ${\lambda }_{c}^{\Omega }$, the superconductivity enters into the intermediate coupling region and the tendencies of ${T}_{c}$ become complicated. Besides, comparing the strong coupling region with the weak coupling region, three tendencies of ${T}_{c}$ are contrasting between the two regions.

Moreover, in the intermediate coupling region (${\lambda }_{c}^{P}<\lambda <{\lambda }_{c}^{\Omega }$), the enhancement methods are more interesting. In particular, take that $\lambda$ belongs in the interval ${\lambda }_{c}^{P}<\lambda <{\lambda }_{c}^{Z}$ for the following discussion without loss of generality. In this interval, superconductivity can be enhanced by increasing $P$ or decreasing $Z$. First, let the superconductivity be optimized by tuning $Z$, such that $\lambda$ is equal to ${\lambda }_{c}^{Z}$, denoted as ${\lambda }_{1}$. Second, since ${\lambda }_{1}$ is larger than ${\lambda }_{c}^{P}$, we can increase $P$ to increase ${T}_{c}.$ The second step causes $\lambda$ to decrease and ${T}_{c}$ is optimized when $\lambda ={\lambda }_{c}^{P}$, denoted as ${\lambda }_{2}$. Third, now ${\lambda }_{2}$ is smaller than ${\lambda }_{c}^{Z}$, ${T}_{c}$ can be further increased by decreasing $Z$. The third step increases $\lambda$ and ${T}_{c}$ is optimized when $\lambda ={\lambda }_{c}^{Z}$, denoted as ${\lambda }_{3}$. Repeat step 2 and step 3 by increasing $P$ and decreasing $Z$ alternately; ${T}_{c}$ can be enhanced like a zigzag mountain climbing. In this study, we propose simultaneously gating and pressurizing on thin-film superconductors to verify our discussion. Furthermore, FeSe has been observed under gating^25^ and pressurizing^50^ independently. More precisely, the negative carrier number effect and the dome-like pressure effect suggest the coupling strength ${\lambda }_{c}^{P}<{\lambda }_{FeSe}<{\lambda }_{c}^{Z}$, which agree with ${\lambda }_{FeSe}=1.6$^47^. Additionally, we propose that increasing $P$ and decreasing $\Omega$ alternately, or increasing $Z$ and decreasing $\Omega$ alternately, are two other methods to enhance superconductivity in the intermediate region.

## Conclusion

In this study, we proposed a phenomenological model based on phonon-mediated interaction, which explains the difference between weak and strong coupling superconductors affected by tuning phonon frequency $\Omega$, carrier number $Z$, and pressure $P$. We introduced the concept of critical coupling constants and enhancement strategies for superconductivity, extending McMillan's results on coupling strength equal to 2. Specifically, the sign of the first-order derivative $d{T}_{c}/d\mathcal{X}$ with respect to $\mathcal{X}=\Omega$, $Z$, or $P$, indicates that ${T}_{c}$ is increasing or decreasing when either these three parameters change. More precisely, these three derivatives have two features in common: (1) the coupling constant $\lambda$ beyond (or below) the critical coupling constant ${\lambda }_{c}^{\mathcal{X}}$ determined $d{T}_{c}/d\mathcal{X}$ to be positive (or negative), and (2) the dome-like delineation observed in strong coupling superconductors because $d\lambda /d\mathcal{X}$ is always negative. Overall, these observations explain the differences between weak and strong superconductors.

Furthermore, using three critical coupling constants ${\lambda }_{c}^{\Omega }$, ${\lambda }_{c}^{Z}$, and ${\lambda }_{c}^{P}$, superconductors can be classified by their coupling strength and consequently, correspond to different enhancement strategies. Specifically, for the weak coupling region ($\lambda <{\lambda }_{c}^{P}$), ${T}_{c}$ can be increased by decreasing $\Omega$, $Z$, and $P$, causing $\lambda$ to be increased, resulting in intermediate coupling. In contrast, superconductors in the strong coupling region ($\lambda >{\lambda }_{c}^{\Omega }$) can be enhanced by increasing $\Omega$, $Z$, and $P$, causing $\lambda$ to be decreased, resulting in intermediate coupling. Moreover, for superconductors in the intermediate coupling region (${\lambda }_{c}^{P}<\lambda <{\lambda }_{c}^{\Omega }$), the zigzag strategies may further enhance superconductivity.

## Data Availability

The datasets used and analyzed during the current study are available from the corresponding author on reasonable request.
